# Lemmel Syndrome: A Surgical Enigma

**DOI:** 10.7759/cureus.65620

**Published:** 2024-07-29

**Authors:** Iqbal M Ali, Saurav K Shetty, Varun Shetty

**Affiliations:** 1 Surgery, Dr. D. Y. Patil Medical College, Hospital and Research Centre, Dr. D. Y. Patil Vidyapeeth (Deemed to be University), Pune, IND

**Keywords:** obstructive jaundice, diverticulitis, cect, duodenal diverticula, lemmel syndrome

## Abstract

A periampullary diverticulum (PAD) is the most common type of duodenal diverticula found in patients undergoing upper gastrointestinal tract assessments. Even while PAD typically does not manifest any symptoms, it can nevertheless be a source of obstructive jaundice in the absence of a tumor or choledocholithiasis, a condition known as Lemmel's syndrome. We report a case of a 60-year-old woman who presented with abdominal discomfort and multiple episodes of vomiting. After comprehensive clinical examination and investigations, a provisional diagnosis of Lemmel's syndrome was made, leading to initial conservative management for diverticulitis, followed by an exploratory procedure. This case underscores the importance of recognizing Lemmel's syndrome as a differential diagnosis for obstructive jaundice when duodenal diverticula are present, to prevent misdiagnosis and ensure timely, appropriate treatment.

## Introduction

Intestinal diverticulosis is a relatively rare condition, often discovered incidentally during examinations for other issues [1]. Within this condition, duodenal diverticula are more frequently observed than those in the jejuno-ileal regions. These diverticula are considered pseudo-diverticula because they lack a muscularis layer and consist solely of mucosal outpouchings [2]. Periampullary diverticula (PAD), which are located within 2-3 cm of the ampulla, are typically asymptomatic [3]. However, these diverticula can occasionally become inflamed and cause complications, particularly involving the pancreas and bile ducts.

A significant complication arising from PAD is Lemmel syndrome. This syndrome is characterized by distal common bile duct (CBD) obstruction resulting from external pressure exerted by a periampullary diverticulum, occurring in the absence of choledocholithiasis or gallstones [2,4]. This external compression can lead to substantial pancreaticobiliary problems, including obstructive jaundice. Obstructive jaundice manifests as a yellowing of the skin and eyes, caused by a bile duct blockage that impedes the normal flow of bile, leading to a buildup of bilirubin in the blood [5]. The mechanical obstruction by the diverticulum triggers these symptoms and necessitates medical intervention.

Management of Lemmel syndrome often involves surgical intervention to alleviate the obstruction caused by the diverticulum. The surgical approach aims to relieve the pressure on the bile duct, thereby resolving the jaundice and related symptoms [6,7]. The case presented in this report involved a patient with obstructive jaundice due to a large periampullary diverticulum. Following surgical decompression, the patient experienced significant improvement, including the normalization of bilirubin levels and resolution of jaundice. This case underscores the critical need for healthcare professionals to recognize PAD as a potential cause of bile duct obstruction and to consider surgical treatment when appropriate to manage this rare but serious condition effectively.

## Case presentation

A 60-year-old female presented to the surgical outpatient department of a tertiary health center with a three-month history of intermittent abdominal discomfort and multiple episodes of bilious vomiting. The patient denied any history of melena, hematochezia, hematemesis, jaundice, fever, or chills. Physical examination revealed only epigastric tenderness. Blood tests showed elevated serum amylase (550 U/L) and lipase (1200 U/L) levels, bilirubin levels (3.2mg/dL), and elevated acute phase reactants such as C-reactive protein(CRP) (23.3 mg/dl). Other biochemical parameters, including alkaline phosphatase, serum glutamic-oxaloacetic transaminase (SGOT), serum glutamate pyruvate transaminase (SGPT), and total leukocyte count, were borderline deranged, while the remaining blood parameters were within normal limits (Table 1).

**Table 1:** Laboratory parameters encountered in our case with their biological reference values

INVESTIGATION	OBSERVED VALUES	BIOLOGICAL REFERENCE VALUES (AS PER OUR INSTITUTIONAL LABORATORY)
Total Bilirubin	3.8 mg/dl	0.22-1.20 mg/dl
Conjugated Bilirubin	3.2 mg/dl	Upto 0.5 mg/dl
Unconjugated Bilirubin	0.6 mg/dl	0.1-1.0 mg/dl
Serum Glutamic-Oxaloacetic Transaminase (SGOT)	54 U/L	8-48 U/L
Serum Glutamate Pyruvate Transaminase (SGPT)	60 U/L	7-55 U/L
Alkaline Phosphatase	135 U/L	40-129 U/L
C-reactive Protein (CRP)	23.3 mg/L	Upto 5.0 mg/L
Serum Amylase	550 U/L	25-115 U/L
Serum Lipase	1200 U/L	8-78 U/L
Hemoglobin (Hb)	14.2 mg/dl	13.2-16.6 mg/dl
Total Leukocyte Count (TLC)	8900/microliter	4000-10000/microliter

**Figure 1:**
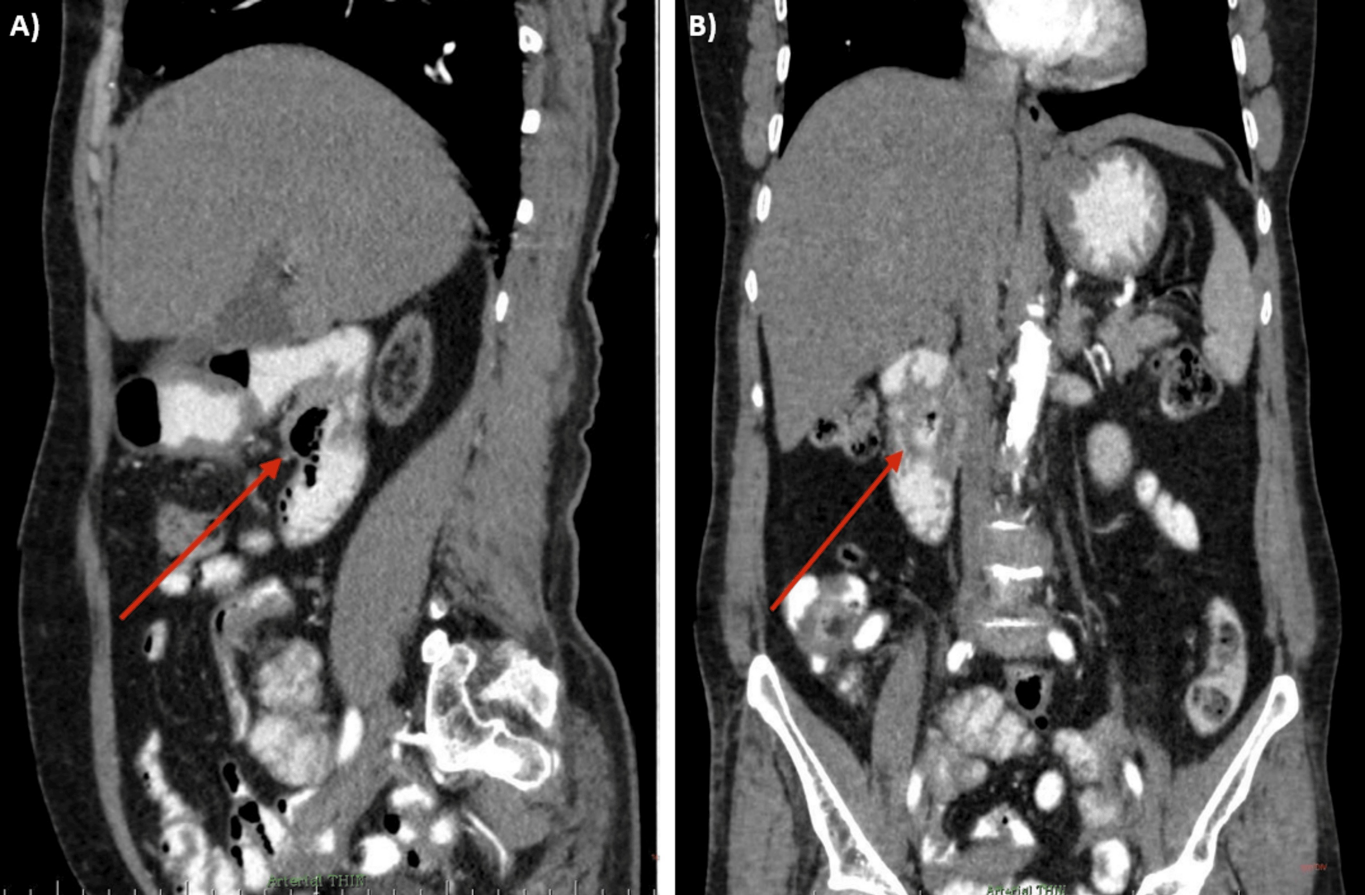
Pre-operative CECT of the abdomen and pelvis The CECT, sagittal (A) and coronal (B) views, show a large per-ampullary diverticulum measuring 4x3 cms (red arrow) causing an obstruction of the distal CBD with upstream dilatation. CECT: contrast-enhanced computed tomography; CBD: common bile duct

A contrast-enhanced computed tomography (CT) scan of the abdomen and pelvis demonstrated a periampullary diverticulum measuring 4x3 cm along the posteromedial aspect of the second part of the duodenum (Figures 1A, 1B). This diverticulum appeared to compress the distal common bile duct (CBD), causing proximal CBD and common hepatic duct (CHD) dilatation. The CT scan also revealed asymmetric thickening of the pyloric region of the stomach, raising suspicion of a neoplastic etiology. To further delineate biliary dilatation, the patient underwent magnetic resonance cholangiopancreatography (MRCP), which confirmed the presence of a large periampullary duodenal diverticulum causing distal CBD compression and proximal CBD/CHD dilatation, along with evidence of diverticulitis. An upper gastrointestinal endoscopy was performed to exclude a pyloric neoplasm, showing chronic gastritis without mass lesions. Biopsies confirmed Helicobacter (H.) pylori-induced gastritis, ruling out neoplastic disease.

**Figure 2:**
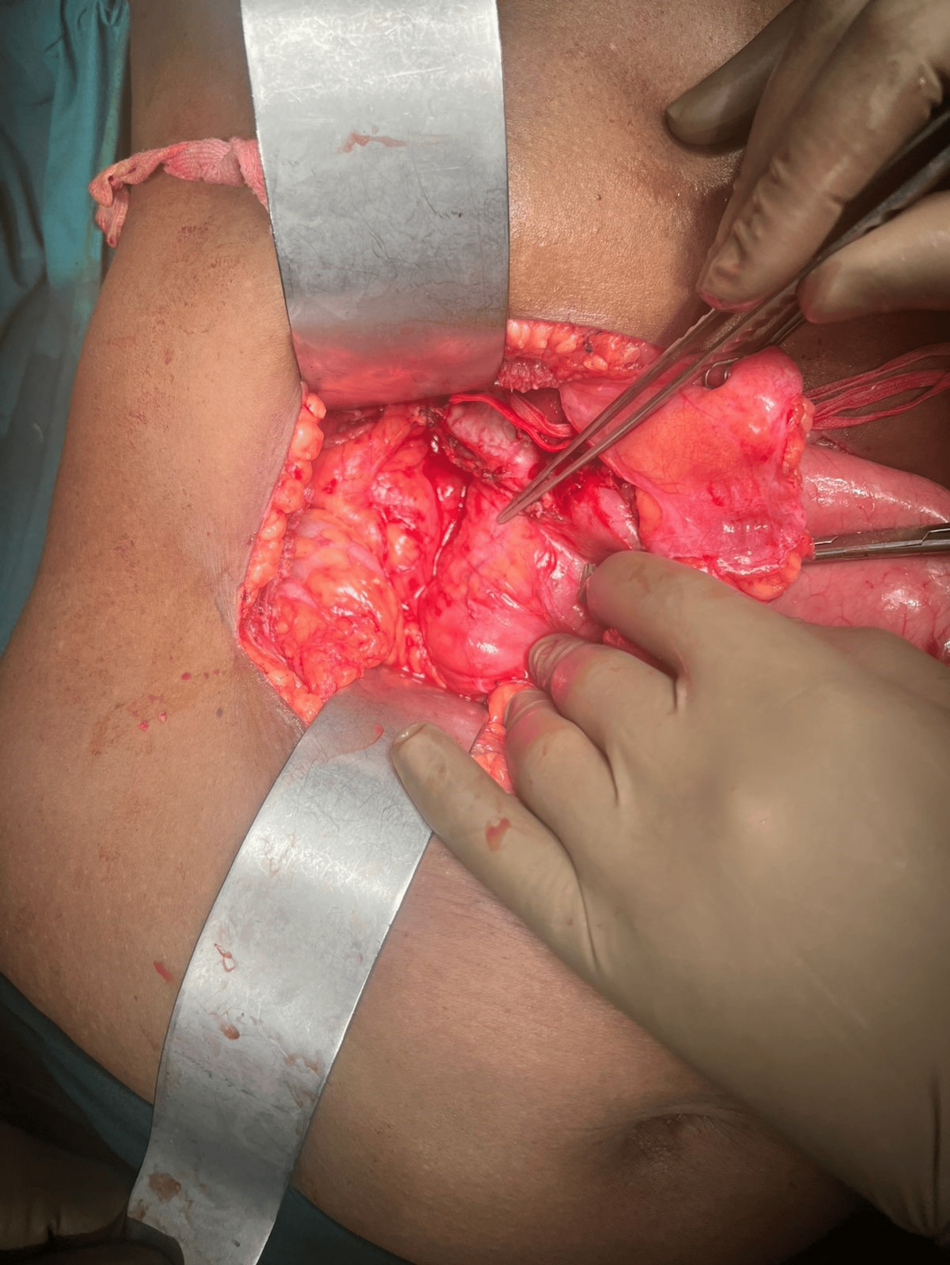
Intra-operative image Intra-operative image showing a periampullary duodenal diverticulum with proximal CBD and CHD dilatation CBD: common bile duct; CHD: common hepatic duct

Given the diagnosis of diverticulitis with pancreatitis, the patient was discharged with a 14-day course of an H. pylori eradication kit. However, she returned to the hospital two weeks later with similar complaints and jaundice. Laboratory investigations revealed conjugated hyperbilirubinemia (Conj. bilirubin: 4 mg/dL) while other parameters remained within normal ranges. An endoscopic retrograde cholangiopancreatography (ERCP) was planned to facilitate stenting of the distal CBD and the pancreatic duct but was unsuccessful due to friable mucosa and bleeding. Surgical intervention was then undertaken. Intraoperative findings included a 4x3 cm diverticulum arising from the posteromedial wall of the second part of the duodenum and partial obstruction at the distal CBD. The patient underwent an isoperistaltic retrocolic posterior gastrojejunostomy, jejunojejunostomy, side-to-side choledochoduodenostomy, and cholecystectomy (Figure 2).

The surgery was uneventful, and the patient had a smooth postoperative recovery. At the three-month follow-up, she was symptom-free, and follow-up blood work showed significant improvement in serum amylase (20 U/L), serum lipase (50 U/L), and CRP (0.9) mg/dl.

## Discussion

Diverticula of the digestive system can appear anywhere along the gastrointestinal tract but are most frequently detected in the colon and duodenum and are protrusions of the intestinal wall [1]. Depending on their location, duodenal diverticula are categorized, with those arising from the periampullary region being the most common type [8,9]. Though PAD seldom cause obstructive jaundice, their prevalence can reach as high as 22% depending on the diagnostic test's sensitivity [10]. Although generally asymptomatic, periampullary diverticula can sometimes cause both pancreaticobiliary and non-pancreaticobiliary complications. Non-pancreaticobiliary complications include diverticulitis, bleeding, perforation, and fistula formation while pancreaticobiliary presentations can range from jaundice and pancreatitis to ascending cholangitis [11]. Lemmel initially defined Lemmel syndrome in 1934 as obstructive jaundice due to a periampullary diverticulum without the presence of gallstones.

Cholangitis, which can be chronic or exacerbate the condition, is caused by the mechanical compression of the terminal bile duct by a diverticulum [12]. Various theories explain the pathophysiology of Lemmel syndrome. First, primary irritation of the ampulla by an inflamed diverticulum can lead to fibrosis and scarring, resulting in jaundice. Second, the sphincter of Oddi may become dysfunctional due to the periampullary diverticula. Third, as in our case, periampullary diverticula can mechanically compress the distal CBD or ampulla [2,4]. Accurate identification and diagnosis of Lemmel syndrome through imaging are crucial to avoid suboptimal care. CT scans and magnetic resonance cholangiopancreatography (MRCP) are vital for diagnosing this syndrome. On these scans, periampullary diverticula, which are small pouches near the ampulla of Vater, may appear as lesions coming from the medial wall of the second part of the duodenum [2]. If these diverticula are fluid-filled, they can be mistaken for a pancreatic pseudocyst or a cystic neoplasm of the pancreatic head [13].

In some clinical situations, the surgical removal of the diverticulum is the best course of action although it is a challenging procedure with a high mortality rate [14]. Excision is possible when there is a biliary blockage; however, for patients with mild symptoms, non-surgical or conservative treatment is advised. Many patients with Lemmel syndrome show symptoms of biliary blockage due to external compression of the common bile duct, necessitating treatment [2,4]. Various treatment options are available, including laparoscopic diverticulectomy, endoscopic diverticulectomy, open diverticulectomy, and endoscopic retrograde cholangiopancreaticography (ERCP) with stenting or bypass procedures [6]. It is crucial to note that not all cases of Lemmel syndrome are linked to periampullary diverticula-induced external compression of the CBD. Treatment options vary based on the underlying etiology and mechanism. Among the many causes of obstructive jaundice, Lemmel syndrome, although rare, should be considered during the differential diagnosis. Radiological and endoscopic evaluations are essential for diagnosing Lemmel syndrome, and treatment modalities range from conservative management to surgical intervention, depending on the condition's severity and underlying pathophysiology.

## Conclusions

Lemmel syndrome, though rare, presents a significant clinical challenge due to its potential to cause obstructive jaundice and associated morbidity. Its diagnosis requires a high degree of clinical suspicion and thorough evaluation, often involving advanced imaging modalities to identify periampullary diverticula accurately. Surgeons and clinicians must consider Lemmel syndrome in the differential diagnosis of obstructive jaundice to facilitate timely intervention and appropriate management, thereby improving patient outcomes and minimizing the risk of complications associated with this condition.
